# Splice Site Mutations in the *ATP7A* Gene

**DOI:** 10.1371/journal.pone.0018599

**Published:** 2011-04-11

**Authors:** Tina Skjørringe, Zeynep Tümer, Lisbeth Birk Møller

**Affiliations:** Department of Applied Functional Human Genetics, The Kennedy Center, Glostrup, Denmark; Auburn University, United States of America

## Abstract

Menkes disease (MD) is caused by mutations in the *ATP7A* gene. We describe 33 novel splice site mutations detected in patients with MD or the milder phenotypic form, Occipital Horn Syndrome. We review these 33 mutations together with 28 previously published splice site mutations. We investigate 12 mutations for their effect on the mRNA transcript *in vivo*. Transcriptional data from another 16 mutations were collected from the literature. The theoretical consequences of splice site mutations, predicted with the bioinformatics tool Human Splice Finder, were investigated and evaluated in relation to *in vivo* results. Ninety-six percent of the mutations identified in 45 patients with classical MD were predicted to have a significant effect on splicing, which concurs with the absence of any detectable wild-type transcript in all 19 patients investigated *in vivo*. Sixty-seven percent of the mutations identified in 12 patients with milder phenotypes were predicted to have no significant effect on splicing, which concurs with the presence of wild-type transcript in 7 out of 9 patients investigated *in vivo*. Both the *in silico* predictions and the *in vivo* results support the hypothesis previously suggested by us and others, that the presence of some wild-type transcript is correlated to a milder phenotype.

## Introduction

Menkes disease (MD) is an X-linked, multisystemic lethal disorder of copper metabolism caused by mutations in the *ATP7A* gene. The severe classical form of MD is characterised by progressive neurodegeneration, connective tissue abnormalities, distinctive “kinky” hair and ultimately death in early childhood. Though most patients (90–95%) exhibit the severe clinical course, there are various milder atypical forms with longer survival of the affected patients (atypical MD). The Occipital Horn Syndrome (OHS), mainly characterized by connective tissue manifestations is the mildest form [1].

A broad spectrum of *ATP7A* mutations (from single amino acid substitutions to large deletions and chromosome aberrations) has been identified in patients with classical or one of the milder MD forms [2]. Approximately 22% of these *ATP7A* mutations are splice site mutations [2].

Splice site mutations are DNA sequence changes that alter or abolish correct mRNA splicing during the process of precursor mRNA maturation. Splice site mutations can result in the complete skipping of one or more exons, retention of introns, creation of a pseudo-exon, or activation of a cryptic splice site within an exon or an intron [3]. The resulting transcript may either be in-frame or out-of-frame. An in-frame transcript may lead to the production of a functional or partially functional protein, whereas out-of-frame transcripts are likely to cause the formation of a premature termination codon and subsequent rapid nonsense–mediated mRNA decay [4], [5]. Some splice site mutations do not abolish the wild-type transcript expression completely, which may lead to less severe phenotypes [6], [7], [8].

This study presents a comprehensive overview of 61 splice site mutations in the *ATP7A* gene including 33 novel mutations, identified in patients with classical MD, atypical MD or OHS. Studies of splice site mutations are highly relevant in order to identify a correlation between the effects of splice site mutations and patient phenotypes, to enable a prediction of the severity of the disease, and to design therapies. It has been proposed that MD patients might especially benefit from early copper therapy when some functional ATP7A protein is present [9]. We investigated the effect of 12 mutations *in vivo*, and 61 mutations with the bioinformatics tool, Human Splicing Finder (HSF). We compared the *in silico* predictions with *in vivo* results and with the observed phenotypes of the patients.

## Materials and Methods

### Samples and mutation detection

The 33 novel splice site mutations were found in a cohort of MD patients who were referred to the Kennedy Center for molecular and/or biochemical diagnosis. Mutations were identified by PCR amplification and direct sequencing of the 23 exons using an ABI3130XL sequencer (Applied Biosystems, Foster, CA).

### Transcript analysis

Fibroblast cell cultures obtained from patient-skin biopsies were grown in a 1∶1 mixture of RPMI 1640 with 20 mM HEPES and a nutrient mixture of F-10 Hams medium, supplemented with 7.5% Amnio Max (Life Technologies), C100 supplement, 4% fetal calf serum, penicillin, and streptomycin. RNA was isolated with RNeasy (QIAgen, Bothell, WA), and single-stranded cDNA was synthesized with the High-Capacity cDNA Archive Kit in accordance with the manufactureŕs instructions (Applied Biosystems, Foster, CA). To investigate the effect of selected splice site mutations, RT-PCR was carried out with primers specific for *ATP7A* and designed to amplify those exon(s) that were expected to be affected by the mutation, and the flanking exons. The cDNA fragment spanning from exon 6 to exon 9 was PCR amplified from 8 patients (cases: 5, 6, 8, 9, 11–14). The cDNA fragment spanning from exon 12 to exon 16 was PCR amplified from patient 32. The cDNA fragment spanning from exon 18 to exon 23 was amplified from 3 patients (cases: 58–60). Primer sequences can be obtained upon request. The PCR products were separated on a 1% agarose gel. The relevant bands were purified, and sequenced using an ABI3130XL sequencer (Applied Biosystems, Foster, CA).

### Bioinformatics

We have compiled 61 *ATP7A* splice site mutations. All were analysed with HSF version 2.4 [10] (http://www.umd.be/HSF/). Only those mutations that are located in the defined splice site consensus sequences are included here. The consensus sequences are (C/A)AG|gt(a/g)agt and cag|G for the donor splice site and the acceptor splice site, respectively [11]. The mutation designations are based on *ATP7A* transcript ENST00000341514 from Ensembl. A in the ATG start codon is defined as c.1. The relative strength of the splice sites obtained from the bioinformatics tool is given as the consensus value (CV), which ranges from 0 to 100. Splice sites with CVs higher than 80 are strong splice sites; less strong sites have CVs that range from 70 to 80. Splice sites with CVs of 65 to 70 are weak, as only a few of these sites are active [10]. Hence, splice sites with CVs below 70 are herein considered non-functional. The CV-output from HSF only indicates the strength of the actual splice site and does not take the possible effect of (and on) other cis-acting elements into account. The effect of a splice site mutation does not solely depend on the CV value, but also on the relative change in ΔCV. Desmet et al. (2009) [10] emphasised that ΔCV reductions of at least 10% for a mutation in any position, or of 7% for a mutation in position +4, are likely to affect splicing.

## Results

When screening the coding region of the *ATP7A* gene in patients with Menkes disease or Occipital Horn Syndrome, we identified a total of 33 novel splice site mutations. They are listed in Table 1 together with 28 previously identified splice site mutations. Forty-five of these mutations lead to classical MD, 6 lead to the less severe atypical MD, and 6 mutations were identified in patients with the mildest phenotype, OHS. Only 4 of the mutations were identified in MD patients with unknown clinical phenotypes.

**Table 1:** *In silico* splice site predictions.

Case (ID)	Pheno-type	Mutation	WT CV	Mutant CV	ΔCV (%)	Exon length variation and CV, if potential cryptic splice site is used	Known transcripts from mutant sequences
1 (93220)	C	c.1707+1G>A (*IVS6, DS*) [14]	82.62	55.79	−32.48	−17: 72.02/+4: 85.11/+16: 70.01/+50: 91.34/+55: 71.12/+86: 87.61	•No wt transcript [14]; •Cryptic splice site leading to 4 bp elongation of exon 6 (frameshift) [14]; •Exon 6 skipping (frameshift) [14]; •Exon 6 and 7 skipping (frameshift)[14]
2 (91209)	C	c.1707+5G>A (*IVS6, DS*) [14]	82.62	70.46	−14.72	−17: 72.02/+16: 70.1/+50: 91.34/+55: 71.12/+86: 87.61	•No wt transcript [14]; •Cryptic splice site leading to 50 bp elongation of exon 6 (frameshift) [14]; •Exon 6 skipping (frameshift) [14]; •Exon 6 and 7 skipping (frameshift) [14]
3 (94268)	O	c.1707+6_+9del TAAG (*IVS6, DS*) [13], [14]	82.62	80.29	−2.83	−17: 72.02/+16: 70.1/+50: 91.34/+55: 71.12/+86: 87.61	•Wt transcript present[13], [14]; •Exon 6 skipping (frameshift)[13], [14]
4 (93279)	C	c.1708-1G>C* (*IVS6, AS*)	89.36	60.42	−32.39	−5: 75.83 (+4.82)/−40: 70.03/−62: 77.13/−64: 70.05/+23: 78.93	
5 (9224)	C	c.1870-1G>C (*IVS7, AS*) [24]	75.73	46.79	−38.22	−2: 91.28 (+12.71)/−7: 80.24 (+3.27)/−16: 74.05/−30: 78.15/−33: 75.45/−43: 73.38/−55: 72.14/+32: 84.74/+62: 71.02	•No wt transcipt*; •Cryptic splice site leading to a 2 bp deletion of exon 8 (frameshift)*
6 (93271)	C	c.1870-2A>T* (*IVS7, AS*)	75.73	46.79	−38.22	−2: 80.88/−7: 80.91 (+4.14)/−16: 74.05/−30: 78.15/−33: 75.45/−43: 73.38/−55: 72.14/+32: 84.74/+62: 71.02	•No wt transcript*; •Cryptic splice site leading to a 7 bp deletion of exon 8 (frameshift)*; •Cryptic splice site leading to a 2 bp deletion of exon 8 (frameshift)*
7 (95222)	C	c.1946+1G>A* (*IVS8, DS*)	83.72	56.88	−32.05	−31: 72.38/−39: 86.07/−53: 74.15/+16:78.3/+65: 84.11	
8 (91266)	C	c.1946+1G>C (*IVS8, DS*) [24]	83.72	56.88	−32.05	−31: 72.38/−39: 86.07/−53: 74.15/+16: 78.3/+65: 84.11	•No wt transcript*; •Exon 8 skipping (frameshift)*
9 (93237)	C	c.1946+1G>T* (*IVS8, DS*)	83.72	56.88	−32.05	−31: 72.38/−39: 86.07/−53: 74.15/+16:78.3/+65: 84.11	•No wt transcript*; •Exon 8 skipping (frameshift)*
10 (91267)	C	c.1946+2T>C (*IVS8, DS*) [15]	83.72	56.88	−32.05	−31: 72.38/−39: 86.07/−53: 74.15/+16: 78.3/+65: 84.11	•No wt transcript[15]; •Exon 8 skipping (frameshift)[15]
11 (94235)	C	c.1946+2_+3del TA (*IVS8, DS*) [24]	83.72	32.58	−61.08	-	•No wt transcript*; •Exon 8 skipping (frameshift)*
12 (94230)	C	c.1946+5G>A (*IVS8, DS*) [24]	83.72	71.55	−14.53	−31: 72.38/−39: 86.07/−53: 74.15/+16: 78.3/+65: 84.11	•No wt transcript*; •Exon 8 skipping (frameshift)*
13 (9529)	C	c.1946+5G>C (*IVS8, DS*) [24]	83.72	71.7	−14.35	−31: 72.38/−39: 86.07/−53: 74.15/+16: 78.3/+65: 84.11	•No wt transcript *; •Exon 8 skipping (frameshift)*
14 (91226)	C	c.1946+6T>G (*IVS8, DS*) [24]	83.72	81.78	−2.31	−31: 72.38/−39: 86.07/−53: 74.15/+16: 78.3/+65: 84.11	•No wt transcript*; •Exon 8 skipping (frameshift)*
15 (92288)	C	c.1947-1G>T* (*IVS8,AS*)	78.85	49.91	−36.71	−6: 71.53 (+3.17)/−24: 79.21/+45: 80.72/+61: 74.54/+68: 82.15	
16 (92285)	A	c.1947-5_-1 dupATAAG (*IVS8,AS*) [15]	78.85	68.06	−13.68	−24: 79.21/+5: 78.85	•No wt transcript [15]; •Cryptic splice site leading to a 5 bp elongation of exon 9 (frameshift) [15]; •Exon 8 skipping and cryptic splice site use in exon 9 (as described above) (in-frame) [15]
17 (94247)	C	c.2171A>C* (*E9,DS*) Gln724Pro	73.16	68.41	−6.49	−16: 73.46/+4: 76.1/+11: 76.23/+59: 71.12/+64: 73.05/+89: 80.17	
18 (92263)	C	c.2172G>T (*E9, DS*) [9] Gln724His	73.16	62.3	−14.85	−16: 73.46/+4: 76.1/+11: 76.23/+59: 71.12/+64: 73.05/+89: 80.17	•No wt transcript[9]; •Exon 9 skipping (frameshift)[9]; •Exon 8 and 9 skipping (in-frame)[9]; •Exon 9 and 10 skipping (frameshift)[9]; •Exon 8 to 10 skipping (in-frame)[9]; •Exon 9 to 10 skipping (in-frame)[9]
19 (96281)	-	c.2172+1G>A* (*IVS9, DS*)	73.16	46.33	−36.68	−16: 73.46/+4: 75.14/+11: 76.23/+59: 71.12/+64: 73.05/+89: 80.17	
20 (96202)	C	c.2172+5G>C (*IVS9, DS*) [20]	76.13	61.15	−16.42	−16: 73.46/+11:76.23/+59: 71.23/+64: 73.05/+89: 80.17	• No wt transcript[20]; • Exon 9 skipping (frameshift)[20]; • Exon 8 and 9 skipping (in-frame)[20]
21 (95237)	A	c.2172+6T>G (*IVS9, DS*) [25]	73.16	71.23	−2.64	−16: 73.46/+11: 76.23/+59: 71.23/+64: 73.05/+89: 80.17	•Wt transcript present[25]; •Exon 9 skipping[25]
22 (95267)	C	c.2172+5_+19delGTGAATTGTTAGCAA* (*IVS9, DS*)	73.16	61	−16.63		
23 (93217)	C	c.2173-1G>C* (*IVS9, AS*)	84.92	55.98	−34.08	−10: 80.46 (+2.3)/−30: 91.77/−58: 73.35/+40: 81.82/+59: 71.26/+67: 75.55	
24 (92276)	-	c.2406G>C* (*E10, DS*) Lys802Asn	84.95	73.93	−12.96	−84: 72.97/+5: 85.11/+15: 78.68/+32: 72.88/+37: 71.5	
25 (94207)	O	c.2406+3A>T (*IVS10, DS*) [16]	84.95	79.92	−5.91	−84: 72.97/+5: 85.11/+15: 78.68/+32: 72.88/+37: 71.5	•No wt transcript[16]; •Exon 10 skipping (in-frame)[16]
26 (91249)	O	c.2497A>G (*E11, DS*) [23] Ser833Gly	78.88	74.02	−6.16	−32: 71.41/−66: 71.28	•Wt transcript present[23]; •Exon 11 skipping[23]; •Cryptic acceptor splice site activation leading to 220 bp deletion[23]
27 (9421)	C	c.2498+1G>A* (*IVS11, DS*)	78.88	52.04	−34.02	−32: 71.41/−66: 71.28	
28 (93242)	C	c.2499-1G>A* (*IVS11, AS*)	83.47	54.52	−34.68	−5: 74.99 (+0.09)/−38: 72.02/−50: 75.22/−53: 72.2/−59: 83.01/−62: 77.07/−74: 80.01/−95: 79.14/+43: 71.15/+48: 76.56/+53: 74.64/+55: 74.32/+68: 73.14/+85: 70.4	
29 (92294)	C	c.2626+2T>A* (*IVS12, DS*)	91.2	64.36	−29.43	−18: 70.02/−22: 73.79	
30	C	c.2626G>C (*E12, DS*) [27] Gly876Arg	91.2	80.18	−12.08	−18: 70.02/−22: 73.79	•No wt protein expression[27]
31 (93233)	C	c.2627-2A>G (*IVS12, AS*) [24]	93.47	64.52	−30.97	−5: 70.12/+43: 76.86/+71: 74.12/+85: 82.75	
32 (91247)	A	c.2627G>A (*E13, AS*) [27] Gly876Glu	93.47	89.31	−4.45	−23: 72.24/−34: 76.68/−39: 80.17/−62: 82.32/−99: 71.17/+43: 76.86/+71: 74.12/+85: 82.75	•Wt transcript present*; •Exon 14 and 15 skipping*
33 (91214)	C	c.2781+1G>A* (*IVS13, DS*)	87.03	60.2	−30.83	−63: 73.43/−88: 78.11	
34 (93243)	C	c.2916+1G>A* (*IVS14, DS*)	84.38	57.55	−31.8	−45: 71.11/−54: 82.53/+4: 74.27/+43: 81.25/+60: 70.37	
35 (9724)	-	c.2916+3_2916 +6delAAGT* (*IVS14, DS*)	84.38	71.21	−15.61	-	
36 (95243)	C	c.2916+5G>A* (*IVS14, DS*)	84.38	72.22	−14.42	−45: 71.11/−54: 82.53/+43: 81.24/+60: 70.37	
37 (94209)	O	c.2917-4A>G (*IVS14, AS*) [21]	87.24	87.17	−0.08	−11: 70.03/−28: 85.47/−55: 78.76/−67: 86.13/+40: 87.11/+71: 75.67	•Wt transcript present[21]; •Exon 15 skipping (in-frame)[21]
38 (96255)	C	c.3111G>T (*E15, DS*) [26] Lys1037Asn	96.71	85.84	−11.24	−29: 75.45/−56: 73.56/−60: 74.47/−68: 70.26/−72: 84.8/+60: 85.64	
39 (94211)	O	c.3111+4A>C* (*IVS15, DS*)	96.71	87.91	−9.1	−29: 75.45/−56: 73.56/−60: 74.47/−68: 70.26/−72: 84.8/+60: 85.64	
40 (95202)	C	c.3112-2A>C* (*IVS15, AS*)	94.41	65.47	−30.66	−6: 79.57 (+3.38)/−24: 71.55/−52: 77.66/−64: 70.27/−69: 72.94/−83: 73.04/−89: 76.04/+35: 71.88/+46: 79.18/+70: 82.84	
41 (92265)	C	c.3112-2A>G* (*IVS15, AS*)	94.41	65.47	−30.66	−6: 77.03 (+0.08)/−24: 71.55/−52: 77.66/−64: 70.27/−69: 72.94/−83: 73.04/−89: 76.04/+35: 71.88/+46: 79.18/+70: 82.84	
42 (92244)	C	c.3294+1G>C* (*IVS16, DS*)	79.03	52.2	−33.96	−50: 74.12/−66: 71.59/+41: 73.71/+71: 72.97	
43	C	c.3293_3294del AG (*IVS16, DS*) [13]	79.03	67.06	−15.15	-	•No wt transcript[13]; •Exon 16 skipping[13]
44 (96201)	-	c.3294+2T>C* (*IVS16, DS*)	79.03	52.2	−33.96	−50: 74.12/−66: 71.59/+41: 73.71/+71: 72.97	
45 (96272)	C	c.3511+1G>A* (IVS17, DS)	87.25	60.41	−30.76	−54: 70.42	
46 (94210)	O	c.3511+5G>A (*IVS17, DS*) [21]	87.25	75.08	−13.94	−54: 70.42	•Wt transcript present[21]; •Exon 17 skipping (frameshift)[21]
47 (95278)	C	c.3658+1delG* (*IVS18, DS*)	92.36	32.29	−65.05	−1: 85.44 (+106.3)	
48 (95219)	C	c.3801+1G>T* (*IVS19, DS*)	87.68	60.85	−30.6	+13: 84.58/+17: 71.34	
49 (93273)	C	c.3801+3A>C* (*IVS19, DS*)	87.68	82.66	−5.73	−54: 79.73/+13: 86.04	
50 (94294)	C	c.3801+4A>G* (*IVS19, DS*)	87.68	79.34	−9.51	+13: 84.58/+17: 71.34	
51	C	c.4004delG (*E20, DS*) [13]	94.19	17.93	−80.96	−1: 99.05 (+86.96)/−66: 76.34/+28: 71.67/+48: 76.3	
52 (91224)	C	c.4005+1G>T* (*IVS20, DS*)	94.19	67.35	−28.49	−1: 79.92 (+50.55)/−66: 76.34/+28: 71.67/+48: 76.3	
53 (92275)	C	c.4005+5G>A* (*IVS20, DS*)	94.19	82.02	−12.92	−66: 76.34/+28: 71.67/+48: 76.3	
54 (96203)	C	c.4006-2A>G (*IVS20, AS*) [15]	82.54	53.6	−35.07	−19: 77.51/−41: 74.39/−43: 73.64/−45: 72.36/−49: 70.58/−56: 70.86/+35: 71.75/+37: 70.96/+41: 74.56/+68: 77.5	•No wt transcript[15]; •Exon 21 skipping (frameshift and premature stop codon)[15]; •Cryptic splice site in exon 21 (frameshift and premature stop codon)[15]
55	C	c.4123+1G>A (*IVS21, DS*) [22]	86.45	59.61	−31.04	−22: 79.55/+4: 73.98/+38: 71.13	
56 (94206)	A	c.4123+3A>T (*IVS21, DS*) [23]	86.45	81.42	−5.81	−22: 79.55/+38: 71.13	•Wt transcript present[23]; •Exon 21 skipping (frameshift)[23]
57 (91284)	A	c.4123+5G>A* (*IVS21, DS*)	86.45	74.28	−14.07	−22: 79.55/+38: 71.13	
58 (92235)	C	c.4226+1G>A* (*IVS22, DS*)	83.43	56.59	−32.17	−85: 77.51/+4: 76.3/+62: 84.93	•No wt transcript*; •Exon 22 skipping*
59 (91233)	C	c.4226+2T>C (*IVS22, DS*) [24]	83.43	56.59	−32.17	−85: 77.51/+4: 78.28/+62: 84.93	•No wt transcript*; •Exon 22 skipping*
60 (9522)	C	c.4226+5G>A* (*IVS22, DS*)	83.43	71.26	−14.58	−85: 77.51/+62: 84.93	•No wt transcript*; •Exon 22 skipping*
61 (92278)	A	c.4226+6T>C* (*IVS22, DS*)	83.43	81.25	−2.61	−85: 77.51/+62: 84.93	

A comprehensive overview of identified splice site mutations in donor sites (DS) and acceptor sites (AS) of the *ATP7A* gene. The mutations are located in exon-intron boundaries in either the intervening sequence (IVS) or in the exon sequence (E). The various mutations lead to different MD phenotypes classified as classical MD (C), atypical MD (A), OHS (O) or unknown (−). The mutations were analysed with the online bioinformatics tool, Human Splicing Finder (HSF), to predict the splicing signals in wild-type and mutated DNA sequences. The strength of the splice sites is indicated by the consensus value (CV) and the CV variation (ΔCV). Potential cryptic splice sites predicted with HSF are given. Effects on pre-mRNA splicing that have been identified *in vivo* are listed.

* Found in this study.

**Figure 1:**
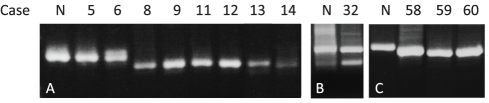
Analysis of *ATP7A* mRNA from patient fibroblasts. RT-PCR was performed on mRNA extracted from 12 different patient fibroblast cultures. The picture is obtained from three different gels marked by space separation. Gel A) Fragment spanning the cDNA sequence from exon 6 to exon 9; Gel B) Fragment spanning the cDNA sequence from exon 12 to exon 16; Gel C) Fragment spanning the cDNA sequence from exon 18 to exon 23. N = Normal, fibroblasts from unaffected individual.

We determined the effect of the mutations on *ATP7A* transcript processing in 12 patients, from whom fibroblast cultures were available (cases: 5, 6, 8, 9, 11–14, 32, 58–60) (Figure 1). In total, the effect on mRNA splicing *in vivo* is described for 28 of the 61 mutations, as the effects of the mutations on mRNA processing in 16 other cases were collected from the literature. The results are summarised in Table 1. Most of the mutations analysed *in vivo* lead to exon-skipping (cases: 3, 8–14, 18, 20, 21, 25, 32, 37, 43, 46, 56, 58–60). Other mutations lead to the use of a cryptic splice site (cases: 5, 6) or to several different transcripts with a combination of exon-skipping and the use of a cryptic splice site (cases: 1, 2, 16, 26, 54). The majority of the reported cases lead to the production of out-of-frame transcripts and hence, probably to non-functional protein. However, in some cases (cases: 3, 21, 26, 32, 37, 46, 56), a limited amount of wild-type transcript is produced alongside the mutant transcript. Only in two cases, leading to atypical MD and OHS respectively (16, 25), wild-type transcript could not be detected. No wild-type transcript was identified in any of the classical MD cases analysed (cases: 1, 2, 5, 6, 8–14, 18, 20, 30, 43, 54, 58–60).

To explore whether a bioinformatics tool can be used to predict the effect of the genomic mutations on mRNA processing, and possibly the severity of the disease, we compared the experimental results and the observed phenotypes with the predictions obtained with the Human Splicing Finder.

The Human Splicing Finder results are listed in Table 1. Seventy-six percent (34/45) of the classical MD cases have a splice site CV below 70. Ninety-three percent (42/45) of the cases have a ΔCV reduction over 10% (or 7% for +4 mutations) (Table 2). Ninety-six percent of the same mutations (43/45) lead to either a CV below 70 or a ΔCV reduction over 10% (or 7% for +4 mutations). Only two of the mutations among the classical MD cases lead to both a CV above 70 and a ΔCV reduction below 10% (cases: 14, 49).

**Table 2:** CV and ΔCV in relation to MD patient phenotypes.

	Total	CV >70	CV<70	ΔCV>10%*	ΔCV<10%*	Prediction of activity of mutant splice site based on the values ΔCV<10%* and CV>70
Classical MD	45 (79%)	11 (24%)	34 (76%)	42 (93%)	3 (7%)	Yes: 2 (4%); No: 43 (96%)
Mild MD	12 (21%)	11 (92%)	1 (8%)	4 (33%)	8 (67%)	Yes: 8 (67%); No: 4 (33%)
All	57	-	-	-	-	-

Using HSF, each mutation is analysed for the effect on the given splice site based on the two parameters CV and ΔCV. Mutated splice sites with CVs above 70 are likely to retain some activity. Conversely, splice sites with CVs below 70 are considered inactive. ΔCV-reductions of less than 10% (or * less than 7% at position +4) are likely to retain some wild-type splice site activity, whereas ΔCV-reductions of more than 10% (7% at position +4) are considered broken and inactive [10]. The mutations are categorized based on the CV- and ΔCV-values obtained in Table 1 “Mild MD” covers atypical MD and OHS phenotypes. Patients with unknown clinical phenotype are not included.

In contrast, among the mutations identified in the 6 atypical MD patients and the 6 OHS patients, only four mutations (33%) lead to a ΔCV reduction over 10% (or 7% for +4 mutations) (cases: 16, 39, 46, 57). One of these mutations (16) also leads to a splice site CV below 70 and, as expected, no detectable wild-type transcript in the fibroblast material. In 67% of the mutations that lead to the mild phenotypes, the splice site CVs were above 70 in combination with ΔCV reductions below 10%, indicating that these sites are active (Table 2). In agreement with this, detectable amounts of wild-type transcript were found in 7 of the 9 analysed patients with mild phenotypes. Only two patients (cases: 16, 25) lacked detectable wild-type transcript.

In 15 cases (2, 12, 13, 17, 24, 30, 35, 36, 38, 39, 46, 50, 53, 57, 60) the CV- and ΔCV-parameters contradicted in the prediction of activity of the splice site. Mutations at the +5 position in particular, are responsible for a large fraction of these cases, as they constitute half of the total inconsistent incidences. In fact, the predictions for 8 out of 9 +5 point mutations are inconsistent. This indicates that the determination of both the CV and the ΔCV is essential.

As splice site mutations may lead to the use of cryptic splice sites, potential splice sites in the vicinity of wild-type splice sites were identified with HSF. Most cryptic splice sites are likely to be located +/− 100 bp on each side of the exon-intron boundary [12]. Therefore only this area was investigated for the presence of potential splice sites. All experimentally identified cryptic splice sites in the patient material were among the potential splice sites suggested by HSF.

## Discussion

This study reviews disease-causing splice site mutations in *ATP7A* in relation to their effect on transcript processing and patient phenotype. Of the 57 mutations identified in patients with known clinical phenotypes, 21% of the mutations lead to the milder phenotypes, atypical MD or OHS (Table 2). We have previously reported that only 9% of all MD patients display the milder phenotypes [2]. Thus, splice site mutations relatively often lead to the milder phenotypes when compared to *ATP7A* mutations in general. This fact renders these mutations highly interesting for further research, and makes predictions of effects on pre-mRNA splicing, using bioinformatics tools, very relevant.

Wild-type transcript was detected *in vivo* in most OHS and atypical MD cases, but not in any of the tested, classical MD cases. We and others have previously shown that even a small amount of wild-type protein may have a major impact on the outcome of the disease in individuals with splice site mutations in *ATP7A* [13], [14]. For instance, 2–5% correctly spliced *ATP7A* mRNA, as compared to the level in unaffected individuals, is enough to develop the mild phenotype, OHS [14].

Assuming that a splice site CV above 70 combined with a ΔCV reduction below 10% (or 7% for +4 mutations) allow the production of some amount of wild-type transcript, whereas a CV below 70 combined with a ΔCV reduction over 10% completely block the production of wild type transcript, 96% of the mutations identified in classical MD patients are predicted to have a significant effect on splicing with no production of wild type transcript. However, this is only the case for 33% of the mutations identified in patients with the milder phenotype. Overall, when using the CV parameter in combination with the ΔCV parameter, 51 of 57 mutations (89%) fit the hypothesis that mild phenotypes develop when wild-type transcript is produced, whereas classical MD is the phenotypic outcome when no wild-type transcript is produced (Table 2). Only, *in vivo* result from one case with classical MD does not fit this hypothesis. In case 14, with a CV above 70 and a ΔCV reduction below 10%, we were, in contrast to the prediction, unable to detect any wild-type transcript *in vivo*. There are also two exceptions, based on *in vivo* results, among cases with the mild phenotypes. In case 16, no wild-type transcript was detected *in vivo* in fibroblasts with the identified mutation, which concurs with the prediction given by HSF. The mild phenotype is thought to be caused by the production of an in-frame transcript, in which exon 8 is skipped and exon 9 is extended with 5 bp [15]; exon 8 encodes the region of the ATP7A-protein between the last metal-binding domain and the first transmembrane domain. In Case 25 with OHS phenotype: we were unable to detect any wild-type transcript; this contradicts the prediction given by HSF. The mild phenotype is also in this case thought to be related to the production of a partially functional protein-variant encoded by an in-frame transcript, which in this case lacks exon 10. The resulting ATP7A-protein lacks transmembrane domains 3 and 4, and is located in the endoplasmatic reticulum instead of its correct location in the trans-Golgi network [16]. It has been proposed that this “mis-localised” protein may retain some of its copper transporting function, resulting in the less severe phenotype.

In approximately one third of the represented exon-intron boundaries (8 of 26) the CV for one or more of the potential splice sites identified with HSF is higher than the CV for the un-mutated wild-type splice site. A similar observation has previously been reported in e.g. the β-globin intron 1 [17]. In β-globin intron 1 the authentic donor splice site is always used in the wild-type sequence, although there is a slightly stronger cryptic splice site close by which is only used when the wild-type splice site is mutationally weakened.

In case 2, HSF found several possible alternative donor splice sites (−17, +16, +50, +55, +86), in the vicinity of the wild-type splice site. We have previously reported that a cryptic splice site at position +50 is used in the mutated sequence in this patient [14]. This means that the active cryptic splice site is not the potential splice site closest to the wild-type splice site. This concurs with observations made by Roca and colleagues [17] who found that 26 of 61 transcripts (43%), in which cryptic splice sites were analysed, skip the nearest potential splice site and use a site further away. The choice of site involves a very complex interplay between various factors, such as sequence context, available transacting factors and secondary structure of the pre-mRNA [17], [18], [19].

In conclusion, both the *in silico* predictions and *in vivo* results support the hypothesis previously suggested by us and others, that the presence of some wild-type transcript correlates with a milder phenotype. Notably, disease-causing mutations identified in patients with mild phenotypes might be difficult to distinguish from benign mutations, which do not cause disease. However, if the family history confirms beyond any doubt, that a splice site mutation is the cause of disease, HSF is very successful in predicting the phenotype.

## References

[1] Tumer Z, Møller LB (2010). Menkes disease.. Eur J Hum Genet.

[2] Møller LB, Mogensen M, Horn N (2009). Molecular diagnosis of Menkes disease: genotype-phenotype correlation.. Biochimie.

[3] Berget SM (1995). Exon recognition in vertebrate splicing.. J Biol Chem.

[4] Silva AL, Romao L (2009). The mammalian nonsense-mediated mRNA decay pathway: to decay or not to decay! Which players make the decision?. FEBS Lett.

[5] Thermann R, Neu-Yilik G, Deters A, Frede U, Wehr K (1998). Binary specification of nonsense codons by splicing and cytoplasmic translation.. EMBO J.

[6] Hubner CA, Senning A, Orth U, Zerres K, Urbach H (2005). Mild Pelizaeus-Merzbacher disease caused by a point mutation affecting correct splicing of PLP1 mRNA.. Neuroscience.

[7] Noordzij JG, de Bruin-Versteeg S, Hartwig NG, Weemaes CM, Gerritsen EJ (2002). XLA patients with BTK splice-site mutations produce low levels of wild-type BTK transcripts.. J Clin Immunol.

[8] Varon R, Dutrannoy V, Weikert G, Tanzarella C, Antoccia A (2006). Mild Nijmegen breakage syndrome phenotype due to alternative splicing.. Hum Mol Genet.

[9] Kaler SG, Buist NR, Holmes CS, Goldstein DS, Miller RC (1995). Early copper therapy in classic Menkes disease patients with a novel splicing mutation.. Ann Neurol.

[10] Desmet FO, Hamroun D, Lalande M, Collod-Beroud G, Claustres M (2009). Human Splicing Finder: an online bioinformatics tool to predict splicing signals.. Nucleic Acids Res.

[11] Mount SM (1982). A catalogue of splice junction sequences.. Nucleic Acids Res.

[12] Nakai K, Sakamoto H (1994). Construction of a novel database containing aberrant splicing mutations of mammalian genes.. Gene.

[13] Gu YH, Kodama H, Murata Y, Mochizuki D, Yanagawa Y (2001). ATP7A gene mutations in 16 patients with Menkes disease and a patient with occipital horn syndrome.. Am J Med Genet.

[14] Møller LB, Tumer Z, Lund C, Petersen C, Cole T (2000). Similar splice-site mutations of the ATP7A gene lead to different phenotypes: classical Menkes disease or occipital horn syndrome.. Am J Hum Genet.

[15] Das S, Levinson B, Whitney S, Vulpe C, Packman S (1994). Diverse mutations in patients with Menkes disease often lead to exon skipping.. Am J Hum Genet.

[16] Qi M, Byers PH (1998). Constitutive skipping of alternatively spliced exon 10 in the ATP7A gene abolishes Golgi localization of the menkes protein and produces the occipital horn syndrome.. Hum Mol Genet.

[17] Roca X, Sachidanandam R, Krainer AR (2003). Intrinsic differences between authentic and cryptic 5′ splice sites.. Nucleic Acids Res.

[18] Caceres JF, Stamm S, Helfman DM, Krainer AR (1994). Regulation of alternative splicing in vivo by overexpression of antagonistic splicing factors.. Science.

[19] Warf MB, Berglund JA (2010). Role of RNA structure in regulating pre-mRNA splicing.. Trends Biochem Sci.

[20] Ogawa A, Yamamoto S, Takayanagi M, Kogo T, Kanazawa M (1999). Identification of three novel mutations in the MNK gene in three unrelated Japanese patients with classical Menkes disease.. J Hum Genet.

[21] Das S, Levinson B, Vulpe C, Whitney S, Gitschier J (1995). Similar splicing mutations of the Menkes/mottled copper-transporting ATPase gene in occipital horn syndrome and the blotchy mouse.. Am J Hum Genet.

[22] Watanabe A, Shimizu N (2005). Identification of three novel mutations in Japanese patients with Menkes disease and mutation screening by denaturing high performance liquid chromatography.. Pediatr Int.

[23] Kaler SG, Gallo LK, Proud VK, Percy AK, Mark Y (1994). Occipital horn syndrome and a mild Menkes phenotype associated with splice site mutations at the MNK locus.. Nat Genet.

[24] Tumer Z, Lund C, Tolshave J, Vural B, Tonnesen T (1997). Identification of point mutations in 41 unrelated patients affected with Menkes disease.. Am J Hum Genet.

[25] Kaler SG, Holmes CS, Goldstein DS, Tang J, Godwin SC (2008). Neonatal diagnosis and treatment of Menkes disease.. N Engl J Med.

[26] Grange DK, Kaler SG, Albers GM, Petterchak JA, Thorpe CM (2005). Severe bilateral panlobular emphysema and pulmonary arterial hypoplasia: unusual manifestations of Menkes disease.. Am J Med Genet A.

[27] Møller LB, Bukrinsky JT, Molgaard A, Paulsen M, Lund C (2005). Identification and analysis of 21 novel disease-causing amino acid substitutions in the conserved part of ATP7A.. Hum Mutat.

